# Innovative Methods for Prenatal Cannabis Exposure: Vapor Inhalation Chamber and Metabolite Quantification in Prairie Voles and Rats

**DOI:** 10.1002/dev.70185

**Published:** 2026-07-24

**Authors:** Sophia Rogers, Casey E. Hogrefe, Chun‐Yi Wu, Adele M. H. Seelke, Anurupa Kar, Sabrina L. Mederos, Jessica Bond, Felisa J. Carbajal, Zhenmo Yu, Melissa D. Bauman, Karen L. Bales

**Affiliations:** ^1^ Department of Psychology University of California, Davis Davis California USA; ^2^ California National Primate Research Center University of California, Davis Davis California USA; ^3^ Department of Neurology and Pharmacology University of California, Davis Davis California USA; ^4^ The MIND Institute, School of Medicine University of California, Davis Sacramento California USA; ^5^ Animal Behavior Graduate Group University of California, Davis Davis California USA; ^6^ Department of Bioengineering, UCLA Samueli School of Engineering University of California, Los Angeles Los Angeles California USA; ^7^ Department of Physiology and Membrane Biology, School of Medicine University of California, Davis Davis California USA; ^8^ Department of Neurobiology, Physiology, and Behavior University of California, Davis Davis California USA

**Keywords:** cannabis, fetal development, prairie voles, prenatal exposure, rats, THC, vaping

## Abstract

The increasing prevalence of cannabis use, including among pregnant women, highlights the critical need for a deeper understanding of prenatal cannabis exposure. This study aimed to develop a standardized cross‐species inhalation exposure protocol to administer the principal psychoactive component of cannabis, Δ9‐tetrahydrocannabinol (THC), to prairie voles (*Microtus ochrogaster*) and laboratory rats (*Rattus norvegicus*), and to investigate the distribution of THC in maternal and fetal tissues following prenatal exposure. Using an established e‐cigarette system for delivering vaporized THC, we administered THC to pregnant prairie voles and rats. THC concentrations were measured in maternal plasma and fetal brain tissue using LC–MS/MS (liquid chromatography coupled with tandem mass spectrometry). We found that THC readily crossed the placental barrier in both species, resulting in significantly higher concentrations of THC in the fetal brain within the THC‐exposed groups compared to the vehicle controls. Interspecies comparison revealed higher THC concentrations in rat fetal brain tissue compared to prairie voles. No significant effects of fetal position on THC levels were found for either species. The findings confirm placental transfer of THC and reveal species‐specific patterns of THC distribution. Additional studies were then carried out in voles to compare plasma and brain THC levels in maternal and virgin adult prairie voles. Maternal brain THC concentrations were significantly higher than fetal brain concentrations in prairie voles. This study establishes a translational model for investigating prenatal cannabis exposure using an aerosolized administration method in voles compared to established methods in rats. The standardized protocol and results provide a foundation for future research into the developmental consequences of prenatal cannabis exposure and offer crucial insights for informing public health policies and clinical practices in response to the global increase in cannabis use.

## 1 Introduction

Cannabis, a plant known for its psychoactive properties, has been used throughout history for various purposes, including medicinal, recreational, and spiritual practices (Russo 2007; Zuardi 2006). The main psychoactive component of cannabis is Δ9‐tetrahydrocannabinol (THC), which interacts with the endocannabinoid system in the body (Mechoulam and Parker 2013). This system consists of endogenous ligands, such as anandamide and 2‐arachidonoylglycerol, which bind to G protein‐coupled cannabinoid receptors (CB1 and CB2) and regulate various physiological processes (Pertwee 2008; Lu and Mackie 2021).

In recent years, the use of cannabis has increased significantly, becoming the third most commonly used psychoactive substance in the United States after alcohol and nicotine, largely due to the legalization of its recreational and medicinal use in many states (Ross and Levy 2023; Hall and Lynskey 2016; Carliner et al. 2017). This increase in cannabis use has also been observed among pregnant women, with higher usage rates in the first trimester compared to the second and third trimesters, and prevalence rates ranging from 3% to 16% across various studies (Volkow et al. 2019; Young‐Wolff et al. 2025a, 2025b; Blair et al. 2025). The use of cannabis during pregnancy raises concerns about the potential effects of prenatal cannabis exposure on fetal development (Jansson et al. 2018). THC readily crosses the placental barrier (Bailey et al. 1987; Calapai et al. 2020), making it important to understand its impact on the developing fetus, particularly during critical developmental periods (Schneider 2009; Rice and Barone Jr. 2000).

A rapidly growing literature in humans gives insight into the effects of prenatal cannabis exposure (Paul et al. 2021; Sorkhou et al. 2024; Nashed et al. 2021; Young‐Wolff, Adams et al. 2024; Avalos et al. 2025; Tadesse et al. 2025), including a greater risk of psychopathology, preterm delivery, low birth weight, neonatal intensive care unit (NICU) admission in newborns, physiological and neurodevelopmental consequences, gastroschisis, omphalocele, and an increased risk of anxiety in offspring. Human studies have explored the developmental, metabolic, and social aspects of cannabis use during pregnancy (Gunn et al. 2016; Hurd et al. 2019; Corsi et al. 2019; Jarlenski et al. 2017). Previous studies have investigated the uptake of other drugs, such as tobacco, during pregnancy (Behnke and Smith 2013; Zhou et al. 2014; O'Connell et al. 2025). Collectively, these studies suggest that social and emotional behavior development might be particularly vulnerable to prenatal cannabis exposure, though underlying mechanisms remain poorly understood.

There is also an expanding number of animal studies showing effects of prenatal and early THC exposures (Iyer et al. 2022; Breit et al. 2022; Hussain et al. 2022; Davies et al. 2025; Roeder et al. 2024; Lei et al. 2023; Lallai et al. 2022), but animal studies examining the effects of cannabis on social behavior, particularly in prairie voles and rat models, are more scarce (Baglot et al. 2022; Frau et al. 2019; Weimar et al. 2020). The work by Baglot and colleagues is one of the few studies on prenatal aerosolized THC administration chamber exposure. Their study demonstrated that prenatal THC exposure in rats led to alterations in social behavior and neural development, highlighting the need for further investigation into the effects of prenatal cannabis exposure in highly social species.

Prairie voles (*Microtus ochrogaster*) and rats (*Rattus norvegicus*) serve as important models for studying the effects of prenatal cannabis exposure due to their well‐characterized social behaviors and developmental processes (McGraw and Young 2010; Bales and Perkeybile 2012). Prairie voles in particular exhibit strong pair bonds and biparental care (Young and Wang 2004), making them an attractive model for investigating the impact of prenatal drug exposure on social development. Rats have been extensively used in developmental neuroscience research (Semple et al. 2013) and provide a well‐established model for studying the effects of prenatal drug exposure on brain development (Schneider 2009). Furthermore, rats engage in rough‐and‐tumble play during their juvenile period, which is crucial for the development of social competence and the refinement of social, emotional, and cognitive skills (Pellis and Pellis 2007).

The primary purpose of the current study was to further validate and build upon a standardized methodology for administering THC to rodent models through an aerosolized administration (“vaping”) chamber, evaluate maternal and fetal THC levels, and expand this model to include a new species, prairie voles. While numerous studies have investigated prenatal cannabis exposure, there are inconsistencies in the literature regarding THC administration methods, with many relying on injections that do not accurately mimic human consumption patterns. Inhalation, particularly combustion inhalation, remains the most common route of administration for cannabis among users, due to its rapid onset of effects (Hindocha et al. 2017; Spindle et al. 2018). Our study aims to develop and validate an aerosolized administration protocol for THC administration in two rodent species: prairie voles and rats. The dosing regimen used in this study was based on previous work by Taffe et al. (2021) and Baglot et al. (2022). This approach more closely resembles human cannabis use, particularly in light of the increasing popularity of aerosolized administration among cannabis users (Budney et al. 2015). By designing and implementing this protocol, we seek to provide researchers with a more translatable method for studying the effects of prenatal cannabis exposure on fetal development.

## 2 Methods

### 2.1 Subjects

#### 2.1.1 Prairie Voles

Prairie voles were descendants of a wild stock originally captured near Champaign, Illinois, and the colony was maintained through systematic outbreeding. The breeding, husbandry, and testing of the prairie voles were conducted at the University of California, Davis. Breeder pairs were housed in large polycarbonate cages (44 × 22 × 16 cm) with aspen wood bedding (Sani‐Chips) and cotton nestlets provided as nesting material. At 20 ± 1 days of age, sexually naïve male and female animals were weaned in groups and subsequently pair‐housed in smaller (27 × 16 × 13 cm) cages with either a same‐sex sibling or an age‐matched, same‐sex non‐sibling. The virgin males and females used in this study were housed in 27 × 16 × 13 cm cages and were initially sexually inexperienced, with no exposure to pups after weaning. Virgin males (*N* = 6) and females (*N* = 6) were dosed to examine the effect of sex and reproductive status on THC absorption. The voles were kept under a 14:10 light–dark cycle, with lights on at 6:00 a.m. They had ad libitum access to water and high‐fiber Purina rabbit chow. The room temperature was maintained at approximately 21°C. The virgin male voles underwent the same procedures as the virgin female voles; however, later statistical analysis revealed no difference between males and females, so the males were dropped from subsequent analyses.

For the fetal exposure study, timed matings were performed to ensure the exact date of conception and, therefore, the precise prenatal exposure day (Kenkel et al. 2019). Sexually mature males and females were placed together in a large cage with cotton for nesting material and a small amount of soiled bedding from the male's cage to act as an olfactory stimulus. Twenty‐three hours later, voles were separated from each other using a plexiglass cage divider to prevent copulation while still allowing for visual, auditory, and olfactory stimulation. The cage divider was removed 2 days later, allowing for mating to occur. This day was considered the day of conception, or embryonic day 0 (E0). A total of four control pairs and six exposed pairs were used for this study.

#### 2.1.2 Rats

Sprague–Dawley lab rats were obtained from Charles River Laboratories. Rats were bred, maintained, and tested at the University of California, Davis. Sexually naïve male and female animals were separated upon arrival into same‐sex housing pairs and allowed to acclimate to the vivarium for approximately 2 weeks. Rats were maintained on a 12:12 light–dark cycle with lights on at 7:00 a.m. Water and food were provided ad libitum. The animals were maintained and observed in large, polycarbonate cages (40 × 30 × 20 cm) with corn cob bedding and paper provided for nesting material. Humidity was controlled, and room temperature was maintained near 21°C. Home cages were left undisturbed beyond weekly cage changes and daily checks of food and water.

After approximately 2 weeks, breeding males were co‐housed overnight with breeding females. Pregnancy was determined the next morning by the presence of a seminal plug. Upon confirmed breeding, females and males were separated, and the males were singly housed. Pregnant females were paired with another female, and pregnancy was monitored by periodic measurements of weight gain. The final group size consisted of six pregnant dams exposed to THC, two pregnant dams exposed to vehicle control only, and two pregnant dams as untreated controls.

### 2.2 Exposure Methods

THC exposure occurred at the California National Primate Research Center (CNPRC) Respiratory Disease Center (RDC) facility. Subjects were moved to the RDC 1 day before the first THC exposure and were temporarily housed within the RDC during the 3 days of THC exposure.

#### 2.2.1 Vapor Chamber Setup and Validation

**FIGURE 1:**
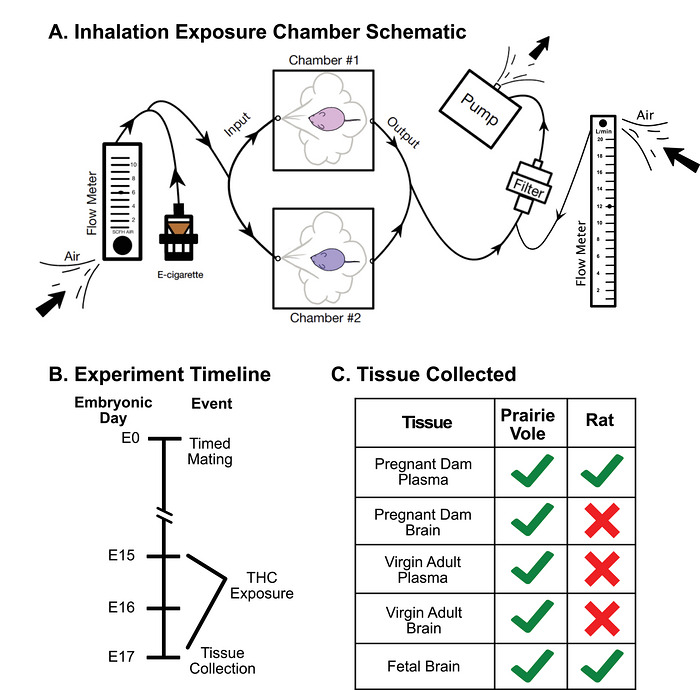
(a) Schematic of apparatus. Atmospheric air is continuously pulled through the apparatus via a pump on the right side of the schematic. Air flow through the system is regulated by two flow meters, and an attached computer controls the heating of the e‐cigarette and the delivery schedule of the vapor. At programmed intervals, the e‐cigarette releases puffs of PG or THC vapor, which fill the sealed exposure chambers. Vapor output flows through a filter and is exhausted through an HVAC filtration system. (b) The timeline of the experiment, including the timed mating of animals and the exposure period from embryonic day 15 (E15) to E17. Tissue collection was conducted on E17, immediately following the exposure period. (c) Endpoints collected for each species.

Subjects were exposed to THC using the two‐chamber T1 tabletop E‐Vape system from La Jolla Alcohol Research, Inc. (Taffe et al. 2021; Nguyen 2020; Glodosky et al. 2020; Gutierrez et al. 2021) (Figure 1A). There were two sizes of exposure chambers. Prairie vole cages fit within the smaller chamber (36.8 × 26.7 × 22.9 cm), while rat cages fit within the larger chamber (52 × 34.3 × 31.8 cm). Each chamber was validated by measuring internal pressure (rats: 18.0–18.5 cm H_2_O, prairie voles: 13.2–13.3 cm H_2_O) and flow rate to ensure consistent and accurate THC delivery to the animals. The flow rate through the chambers was measured using a mini‐BUCK Primary Flow Calibrator. The flow rate for the smaller prairie vole chambers was measured at 12.34 L/min (flow rate of 6.17 L/min for each chamber). The flow rate for the bigger rat chambers was measured at 11.5 L/min (flow rate of 5.75 L/min for each chamber). Flow rates were limited using two flow meters, one regulating air input to the chamber at a flow rate of 6 standard cubic feet per hour (SCFH) of air, and one regulating the air flow from the chamber to the output pump at 12 L/min.

#### 2.2.2 THC E‐Liquid Preparation

THC was received from the National Institute on Drug Abuse (NIDA) Drug Supply Program (NDSP) as 200 mg/mL delta9‐tetrahydrocannabinol (THC) in 95% ethanol solution. The THC was prepared for use in the E‐Vape system by evaporating the ethanol under a stream of nitrogen gas, resulting in a concentrated THC resin. The resin was dissolved in propylene glycol (PG) to a total volume twice that of the original THC–ethanol solution to achieve a concentration of 100 mg/mL. The mixture was vortexed and placed in a lukewarm water bath to ensure complete suspension. The final volume and composition of the THC–PG solution were recorded, and the mixture was wrapped in plastic wrap and foil and stored in a refrigerator at 4°C until use. The e‐liquid solution was brought to room temperature prior to use and loaded into the e‐cigarette (Smok Baby Beast Brother V8 X‐Baby Q2).

#### 2.2.3 Vapor Chamber Experiment

The timeline of the experiment is depicted in Figure 1B. Both prairie voles and rats were dosed over 3 consecutive days, from embryonic day 15 to 17 (E15–17) of the 21‐day gestation. Each exposure session lasted 15 min with one 5‐s puff of vapor delivered every 2 min, with eight puffs in total (Baglot et al. 2022). Experimental subjects were exposed to puffs of 100 mg/mL THC/PG solution, while the vehicle subjects were exposed to puffs of PG only. Untreated control rat subjects (*N* = 2) were used as a baseline and were not placed in the apparatus or exposed to PG solutions. An outline of experimental approaches and species‐specific outcomes is summarized in Figure 1C.

#### 2.2.4 Tissue Collection

**FIGURE 2:**
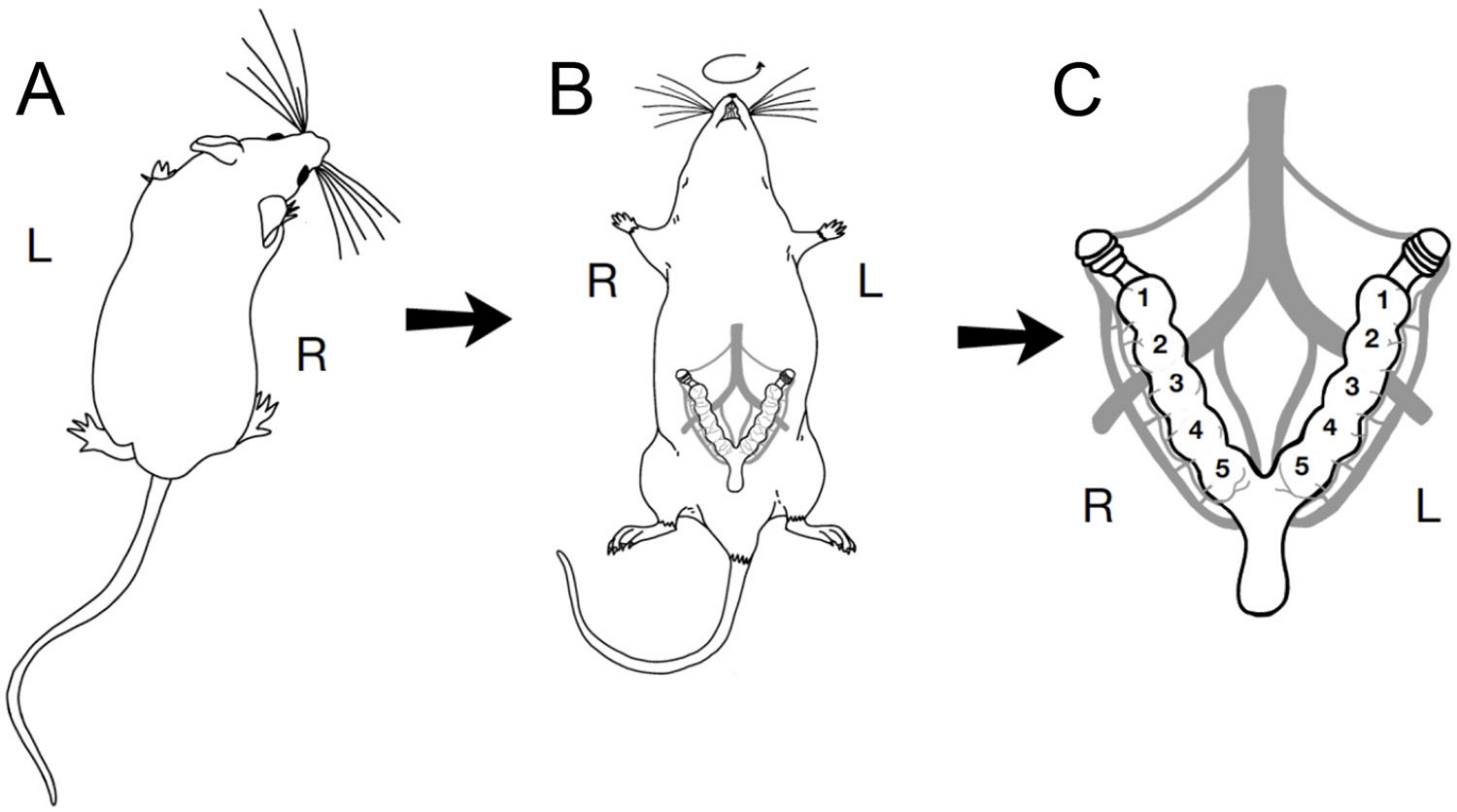
Illustration of rodent uterine anatomy. Immediately following the final session of THC exposure, dams were euthanized, and fetal and maternal tissues were collected. (a) Dorsal view of a behaving rodent, with left and right sides indicated. (b) Rat and vole dams were placed supine to allow for the collection of fetal tissue. The relative position of the uterus is shown on the ventrum, and the left and right sides of the animal are maintained relative to the dorsal view. (c) Detailed schematic of the anatomy of rodent uterine horns and fetal positions. The ovaries are found at the rostral end of the uterine horns, while the vaginal canal is found at the caudal point where both horns join. Fetal position is defined by placement relative to the ovaries, with “1” being the position closest to the ovary and progressively higher numbers indicating greater distance from the ovary. Fetal subjects were given a unique identifier based on both the uterine horn (left vs. right) and position within the horn.

Immediately following THC exposure on Day 3, the exposed pregnant females in both species were anesthetized using isoflurane and euthanized via cervical dislocation followed by rapid decapitation. Maternal blood was collected and centrifuged to obtain plasma, which was aliquoted and stored at −20°C. Maternal vole brains were also collected and flash‐frozen using dry ice. Virgin vole plasma and brain were also collected using the same procedure outlined. Fetal tissues were collected, and their uterine position was recorded (Figure 2). Fetal brains, placentas, and tissue were collected, flash‐frozen using powdered dry ice, wrapped in aluminum foil, and stored in glass scintillation vials at −80°C. Prairie vole fetal tissue was used for later sex determination via PCR analysis. All fetuses were viable at the time of collection.

Detailed values for exact quantities of solution used, THC exposure, and the number of animals, pups, and dams dosed across groups are summarized in Tables 1 and 2.

**TABLE 1:** THC use per day. Mean propylene glycol (PG) solution used per chamber (g) across three exposure days (Days 1–3), along with mean THC exposure per chamber (mg), for prairie voles and rats in THC and control groups.

		Mean PG solution used per chamber (g)			Mean THC exposure per chamber (mg)		
		Day 1	Day 2	Day 3	Day 1	Day 2	Day 3
Prairie vole	THC	0.44 ± 0.02	0.46 ± 0.02	0.43 ± 0.07	42.23 ± 1.76	44.76 ± 1.63	41.70 ± 6.49
	Control	0.53 ± 0.01	0.53 ± 0.01	0.50 ± 0.01	—	—	—
Rat	THC	0.73 ± 0.07	0.60 ± 0.05	0.55 ± 0.04	70.29 ± 6.52	57.8 ± 4.81	52.6 ± 3.91
	Control	0.55 ± 0	0.53 ± 0	0.54 ± 0	—	—	—

*Note:* Data represent mean ± SE.

**TABLE 2:** Dam weight per day. Mean dam body weight (g) across three days for prairie vole and rat THC and control groups, along with the mean number of fetuses per dam in each group.

		Mean dam body weight (g)			Mean # fetuses
		Day 1	Day 2	Day 3	
Prairie vole	THC	50.69 ± 2.69	50.42 ± 2.83	51.43 ± 2.59	4.00 ± 0.45
	Control	47.53 ± 2.37	52.45 ± 2.43	53.83 ± 2.57	4.25 ± 0.48
Rat	THC	293.83 ± 6.14	305.23 ± 4.88	317.47 ± 5.79	14.17 ± 1.40
	Control	304.40 ± 11.00	316.40 ± 13.40	330.70 ± 11.50	16.00 ± 0.91

*Note:* Data represent mean ± SE.

#### 2.2.5 Genotyping for Prairie Vole Fetal Sex

Genomic DNA (gDNA) was extracted from fetal body tissues using a solution containing 25 mM NaOH and 0.2 mM EDTA, followed by neutralization with 40 mM Tris‐HCl. PCR amplification was subsequently performed using specific primers targeting the male‐specific SRY gene to determine the sex of the fetus. The PCR products were analyzed by agarose gel electrophoresis to confirm the presence of the SRY gene fragment, indicative of male sex. The sequences for SRY primers for the Prairie Voles were as follows:

forward 5′‐TTATGCTGTGGTCTCGTGGTC‐3′, and
reverse 5′‐GCA GTCTCTGTGCCTCTTGG‐3′

#### 2.2.6 Quantification of THC and Its Metabolites in Plasma and Brain

Plasma and brain tissue samples were analyzed for THC and its metabolites by the UC Davis Bioanalysis and Pharmacokinetics Core Facility.

### 2.3 Plasma Samples

THC and its metabolites, 11‐hydroxy‐Δ9‐tetrahydrocannabinol (11‐OH‐THC) and 11‐NOR‐9‐carboxy‐Δ9‐tetrahydrocannabinol (11‐NOR‐9‐COOH‐THC), in prairie vole and rat plasma calibrators (0.5, 1, 5, 10, 50, 100, and 500 ng/mL of each standard diluted in blank prairie vole and rat plasma with K2DTA), quality control (QC) samples (1, 10, and 100 ng/mL of each standard diluted in blank prairie vole and rat plasma), and study samples were extracted by mixing 20 µL of the plasma with 80 µL of an internal standard solution that contained 100 ng/mL of each of D_3_‐THC, D_3_‐11‐OH‐THC, and D_9_‐11‐NOR‐9‐COOH‐THC in methanol (MEOH). All standards were purchased from Sigma–Aldrich (St. Louis, MO). The resulting precipitants were removed by centrifugation at 17,000 × *g* at room temperature for 5 min. Each supernatant was transferred to 96 well plate, and 5 µL was injected for LC–MS/MS analysis with Waters (Milford, MA) Acquity I‐class ultra‐performance liquid chromatography (UPLC) hyphenated with Waters Xevo TQ‐S tandem triple quadrupole mass spectrometry (MS/MS). LC–MS‐grade water (H_2_O) with 0.1% formic acid (FA) was used as mobile phase A, and acetonitrile (ACN) with 0.1% FA was used for mobile phase B. All solvents and reagents were purchased from Fisher (Waltham, MA). The following UPLC linear gradient was used to resolve and elute the compounds: 0–0.25 min 40% B; 1.25 min 42.5% B; 2.00 min 50% B; 3.75 min 95% B; 3.75–4.25 min 95% B; 4.26 min 40% B; and 4.26–5.50 min 40% B. The flow rate was maintained at 0.4 mL/min. Mobile phase A was also used for purging, and 50/50 (v/v) ACN/H_2_O was used for needle wash. Phenomenex (Torrance, CA) Kinetex 1.7 mm C18, 100 Å, 100 × 2.1 mm column was used to separate the compounds. The column temperature was maintained at 30°C, and the autosampler temperature was maintained at 10°C. The eluted compounds were fed to the MS/MS and were first ionized with electrospray ionization at positive mode (ESI+). Those parent ions were further fragmented into daughter ions. The following multiple reaction monitoring (MRM) mode was applied to specifically monitor the fragmentation of each compound for the quantification: THC: 315.3 → 193.1; 11‐OH‐THC: 331.3 → 193.1; 11‐NOR‐9‐COOH‐THC: 345.2 → 193.1; D_3_‐THC: 318.3 → 196.1; D_3_‐11‐OH‐THC: 334.3 → 196.2; and D_9_‐11‐NOR‐9‐COOH‐THC: 354.3 → 196.8. The FDA Bioanalytical Method Validation Guidance for Industry was followed to perform QC for both rat and prairie vole plasma calibration curves. The lower limit of quantification (LLOQ) QC of each test article in the corresponding matrix passed the <20% deviation (from nominal concentration) passing criteria, and the rest of the QCs at all concentration levels passed the <15% deviation passing criteria. The rat plasma calibration curve also passed QC with prairie vole plasma QC samples. Therefore, rat plasma can be the surrogate matrix for prairie vole plasma, and rat calibration curves were then used to quantify both rat and prairie vole samples.

### 2.4 Brain Samples

THC and its metabolites in prairie vole and rat brains were quantified using the same LC–MS/MS method described above. The brains were weighed and homogenized in SPEXSampleprep (Now Cole‐Parmer, Metuchen, NJ) grinding tubes pre‐filled with either 2.8‐mm stainless‐steel beads or 3‐mm zirconium beads with a two‐ to fivefold volume of H_2_O, assuming that the density of the brain and water is equal. The tubes were shaken at 1750 rpm in SPEXSampleprep Geno/Grinder2010 for 1 min at room temperature three times, with a 15‐s break in between each cycle. For making the brain homogenate calibrators and QC samples, the blank brains were homogenized with H_2_O, and the brain homogenates were spiked with standard THC and metabolite solutions to make brain homogenate calibrators (0.5, 1, 5, 10, 50, 100, and 500 ng/mL of each standard in blank prairie vole and rat brain homogenates), and QC samples (1, 10, and 100 ng/mL of each standard diluted in blank prairie vole and rat brain homogenates). Forty microliters of each homogenate were mixed with 160 µL of the internal standard solution in methanol as mentioned above. After centrifugation at 17,000 × *g* at room temperature for 5 min, each resulting supernatant was transferred to a 96‐well plate, and 5 µL was injected for LC–MS/MS analysis. Each rat and prairie vole brain calibration curve passed QC at all concentration levels of QC samples in each corresponding matrix. Rat brain calibration also passed QC with prairie vole brain QC samples. Therefore, rat brain homogenate can be the surrogate matrix for prairie vole brain homogenate, and rat brain calibration curves were then used to quantify both rat and prairie vole brain samples.

### 2.5 Statistical Analysis

All statistical analyses were conducted using RStudio (Version 2023.12.1+402; R Core Team 2023). Data were first assessed for normality using the Shapiro–Wilk test. When data did not meet normality assumptions, nonparametric tests were performed. Group differences were analyzed using the Wilcoxon rank‐sum test. For fetal‐related statistical analyses, litter size was included as a fixed‐effect covariate, while litter identity was included as a random effect to account for nonindependence of pups within litters. Relationships between variables were evaluated using Pearson's product–moment correlation (*cor.test*). Linear mixed‐effects models were fitted using the *lme4* package. Statistical significance was defined as *p* < 0.05. Figures were generated using GraphPad Prism.

## 3 Results

### 3.1 Physical and Behavioral Observations

During dosing, all subjects initially showed increased locomotion in the novel chamber. The control animals continued this increased locomotor behavior throughout the exposure period. Approximately 2 min after the initial puff, the dosed dams showed a marked decrease in locomotor activity combined with slumped posture and closed eyelids. The mean volume of PG vehicle and THC administered during each day of testing for each species and group is shown in Table 1. The mean body weight and number of fetuses present in each species and group are shown in Table 2.

### 3.2 Prairie Voles

#### 3.2.1 Prairie Vole Pregnant Dam Plasma

**FIGURE 3:**
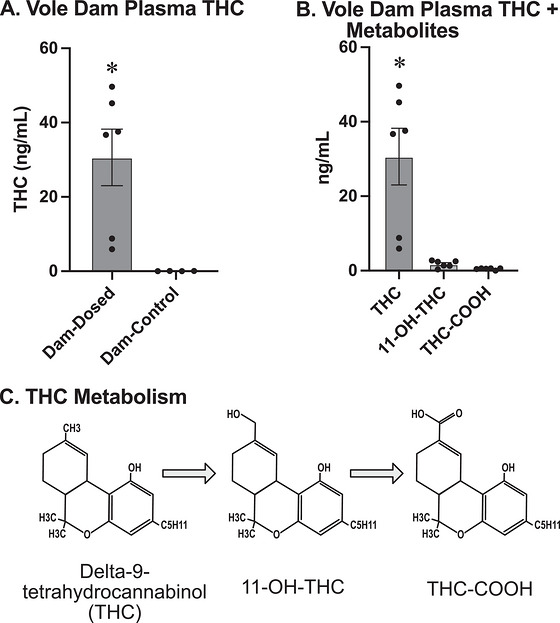
(a) Concentration of THC (measured in ng of THC per mL of tissue) in the plasma of dosed prairie vole dams (left) and vehicle prairie vole dams (right). The plasma of dosed dams contained significantly higher concentrations of THC than vehicle dams. (b) THC and metabolites in the dosed prairie vole dam plasma. THC‐COOH concentration (right) and 11‐OH‐THC concentration (center) are both significantly lower than THC (left). (c) Metabolic progression of the THC molecule. Delta‐9‐tetrahydrocannabinol (THC) is first metabolized into 11‐hydroxy‐delta‐9‐tetrahydrocannabinol (11‐OH‐THC), then further metabolized into 11‐NOR‐9‐carboxy‐delta‐9‐tetrahydrocannabinol (THC‐COOH). Ultimately, this THC‐COOH is conjugated with glucuronide, forming a water‐soluble compound that can be easily excreted from the body. The asterisk (*) indicates that the value is significantly different from all other groups.

The Shapiro–Wilk test revealed a nonnormal distribution of THC concentrations in plasma (*W* = 0.78519, *p* = 0.009576) of vehicle versus THC‐exposed dam prairie voles. A Wilcoxon rank‐sum test showed a significant difference between dosed dam THC concentrations and vehicle dam THC concentrations for plasma (*W* = 24; *p* = 0.01421; Figure 3A). The average amount of plasma THC for dosed dam prairie voles was 30.65 ng/mL. For the prairie vole dam plasma, we also measured the two major metabolites of THC, 11‐OH‐THC and 11‐NOR‐9‐COOH‐THC (THC‐COOH) (Figure 3C). There was a significant difference between dosed dam and vehicle dam concentrations for 11‐OH‐THC (*W* = 24, *p* = 0.01306) and THC‐COOH (*W* = 22, *p* = 0.03073) in plasma. The concentrations of the metabolites in pregnant dosed dams were significantly lower when compared to THC plasma concentrations for both 11‐OH‐THC (estimated −28.865, *p* = 0.000327; Figure 3B) and THC‐COOH (estimated −30.181, *p* = 0.000216). There was a significant difference between the 11‐OH‐THC concentration and the THC‐COOH concentration for the dam prairie vole plasma (*V* = 21, *p* = 0.03603).

#### 3.2.2 Prairie Vole Fetal Brain

**FIGURE 4:**
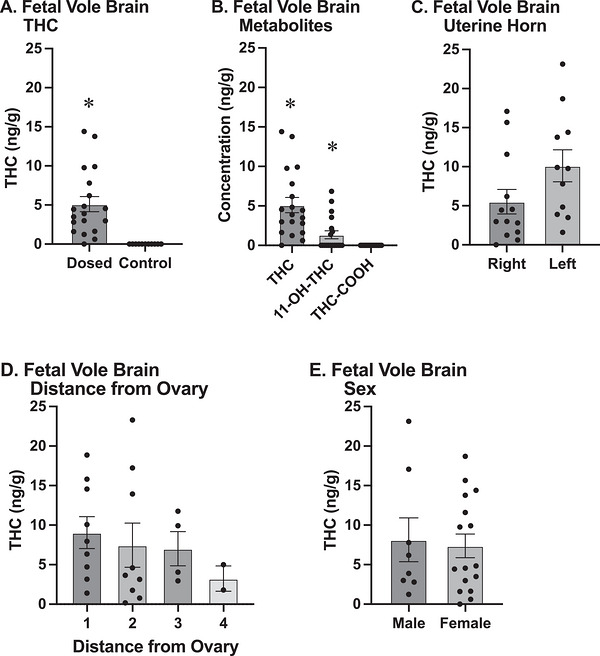
(a) THC concentration (measured in ng of THC per g of tissue) in the brains of dosed (left) versus control (right) fetuses. There was significantly higher THC concentration in the brains of dosed subjects compared to control subjects, and no THC was detected in the brains of any of the control subjects. (b) THC and metabolites in the fetal brain. Both THC (left) and 11‐OH‐THC (center) were found at significantly higher levels than COOH‐THC (right) in the brains of fetuses dosed with THC. (c) The concentration of THC in the brains of vole fetuses found in the left uterine horn (right) did not differ from that in the right uterine horn (left). (d) Fetal position in the uterine horns was identified based on distance from the ovary, with 1 being adjacent to the ovary and higher numbers being progressively closer to the vaginal canal. THC concentration in fetal vole brains did not differ based on position within the uterine horn. (e) THC concentration in the brains of dosed fetuses in relation to sex. There was no difference in THC concentration in the brains of male and female fetal subjects. The asterisk (*) indicates that the value is significantly different from all other groups.

THC levels showed nonnormal distribution (*W* = 0.75316; *p* = 6.455 × 10^−7^) and were found to be significantly higher in dosed prairie vole fetuses compared to vehicle prairie vole fetuses (*W* = 399.5, *p* = 6.934 × 10^−8^; Figure 4A). Across litters, the prairie vole fetal brain THC levels averaged ∼7.6 ng/g, compared with ∼30.6 ng/mL detected in the dam plasma, indicating approximately 25% of maternal plasma concentrations.

In addition to THC, we measured its two major metabolites (Figure 4B), 11‐OH‐THC and THC‐COOH; their presence shows that not only is THC present, but it is also being actively metabolized by the body. A Wilcoxon rank‐sum test showed a significant difference in 11‐OH‐THC concentration between the dosed and vehicle fetal prairie vole brains (*W* = 306; *p* = 0.0008349). The THC metabolite THC‐COOH was not detectable in any fetal prairie vole brains; since both groups contained only zeros, no statistical comparison was possible. A linear mixed model revealed that for dosed fetal samples, accounting for litter size and litter effects, the concentrations of 11‐OH‐THC and THC‐COOH were significantly lower than that of THC brain concentrations, with estimated differences of −5.69 ng/g (*p* = 4.36 × 10^−8^) and −7.63 ng/g (*p* = 8.34 × 10^−12^). Further analysis revealed a significant difference between the concentrations of 11‐OH‐THC and THC‐COOH in dosed fetal prairie vole brain tissue when accounting for litter size and litter effects (estimate = −1.9413, *p* = 7.85 × 10^−6^). Litter size was not a significant predictor of fetal brain concentrations in the fetal metabolite comparisons.

Fetal position analysis revealed no significant differences in brain THC concentrations based on uterine horn side (*t* = −1.377; *p* = 0.1844; Figure 4C) or specific position within the horn (*t* = −0.481; *p* = 0.6368; Figure 4D) after accounting for individual dam differences.

### 3.3 Rats

#### 3.3.1 Rat Pregnant Dam Plasma

**FIGURE 5:**
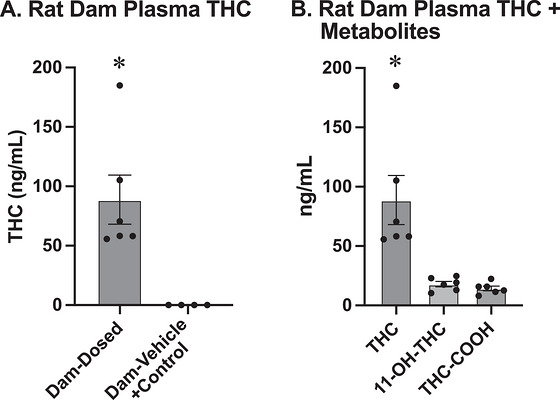
(a) Concentration of THC in the plasma of dosed rat dams (left) and vehicle + control rat dams (right). The plasma of dosed rat dams contained significantly higher concentrations of THC than vehicle + control rat dams. (b) THC and metabolites in the dosed rat dam plasma. THC‐COOH concentration (right) and 11‐OH‐THC concentration (center) are both significantly lower than THC (left). The asterisk (*) indicates that the value is significantly different from all other groups.

THC concentrations within the plasma of vehicle and untreated control dam rats were uniformly zero, and no statistical comparison could be made, since there was no variability in the data. Subsequent analyses combined both vehicle and untreated control dam rat groups into a vehicle control group. A Shapiro–Wilk test revealed a nonnormal distribution of THC concentrations in rat dam plasma (*W* = 0.8381; *p* = 0.04187) of vehicle versus THC‐exposed rats. A Wilcoxon rank‐sum test showed a significant difference between dosed dam THC concentrations and vehicle control dam THC concentrations for plasma (*W* = 24, *p* = 0.01392; Figure 5A). The average amount of plasma THC for dosed dam rats was 88.78 ng/mL. For the rat dam plasma, we also measured 11‐OH‐THC and THC‐COOH. For the dams, there was a significant difference between dosed dam and vehicle control dam concentrations for 11‐OH‐THC (*W* = 24, *p* = 0.01392) and THC‐COOH (*W* = 24, *p* = 0.01142). Concentrations of the metabolites were significantly lower when compared to THC plasma concentrations for both 11‐OH‐THC (estimated −70.81, *p* = 0.000855; Figure 5B) and THC‐COOH (estimated −74.42, *p* = 0.000559). There was not a significant difference between the 11‐OH‐THC concentration and the THC‐COOH concentration for the dam rat plasma (*V* = 19, *p* = 0.09349).

#### 3.3.2 Rat Fetal Brain

**FIGURE 6:**
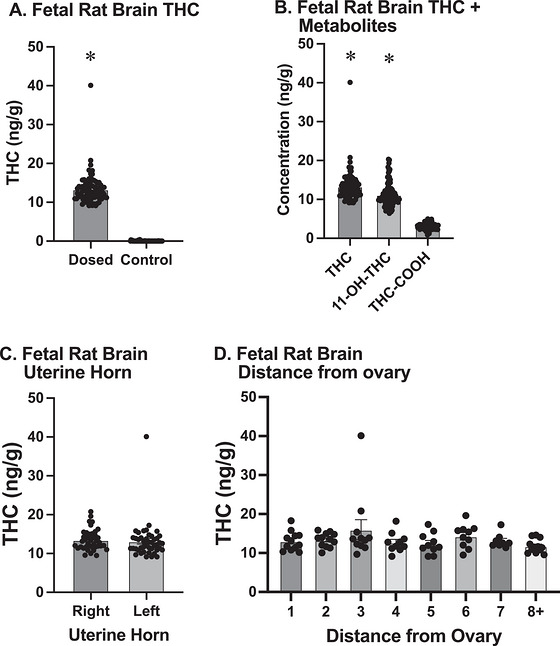
(a) THC concentration (measured in ng of THC per g of tissue) in the brains of dosed (left) versus control (right) rat fetuses. There was significantly higher THC concentration in the brains of dosed subjects compared to control subjects, and no THC was detected in the brains of any of the control subjects. (b) THC and metabolites in the fetal brain. Both THC (left) and 11‐OH‐THC (center) were found at significantly higher levels than COOH‐THC (right) in the brains of fetuses dosed with THC. (c) The concentration of THC in the brains of rat fetuses found in the left uterine horn (right) did not differ from that in the right uterine horn (left). (d) Fetal position in the uterine horns was identified based on distance from the ovary, with 1 being adjacent to the ovary and higher numbers being progressively closer to the vaginal canal. THC concentration in fetal rat brains did not differ based on position within the uterine horn. The asterisk (*) indicates that the value is significantly different from all other groups.

THC concentrations in fetal rat brain tissue showed nonnormal distribution (*W* = 0.80358, *p* = 9.125 × 10^−13^). THC concentrations for vehicle and control fetal rat brain tissue were not significantly different (*p* = 0.4336) and were also combined into a vehicle control group for subsequent analyses. Dosed fetal rats exhibited significantly higher brain THC concentrations compared to vehicle control fetal rats (*W* = 0; *p* ≤ 2.2 × 10^−16^; Figure 6A). Dosed fetal rats exhibited significantly higher brain THC concentrations compared to vehicle control fetal rats (*W* = 0; *p* ≤ 2.2 × 10^−16^; Figure 6A). Mean rat fetal brain THC levels were approximately 13.33 ng/g while maternal plasma levels averaged ∼ 88.78 ng/mL, indicating that fetal rat brain exposure amounted to roughly 15% of dam plasma levels.

In addition to THC, we measured its two major metabolites in fetal rats. A Wilcoxon rank‐sum test showed a significant difference in 11‐OH‐THC concentration between the dosed and vehicle fetal rat brains (*W* = 0; *p* ≤ 2.2 × 10^−16^). A Wilcoxon rank‐sum test showed a significant difference in THC‐COOH concentration between the dosed and vehicle fetal rat brains (*W* = 3; *p* ≤ 2.2 × 10^−16^). A comparison using linear mixed models was conducted to examine the concentrations of THC and its metabolites, 11‐OH‐THC and THC‐COOH, for dosed rat fetal samples and indicated that the concentrations of 11‐OH‐THC (estimate = −2.1229, *p* = 2.68 × 10^−7^; Figure 6B) and THC‐COOH (estimate = −10.2653, *p* ≤ 2 × 10^−16^) were significantly lower than that of THC brain concentrations, when accounting for litter size and litter effects. Litter size was not a significant predictor of fetal brain concentrations. The concentrations of 11‐OH‐THC and THC‐COOH in fetal rat brain tissue were significantly different from each other, with THC‐COOH lower than 11‐OH‐THC (estimate = 8.1424, *p* ≤ 2 × 10^−16^).

No significant difference was found between left and right uterine horn sides (*p* = 0.949; Figure 6C) or between specific positions within the horn (*p* = 0.579; Figure 6D), accounting for individual dam variability.

#### 3.3.3 Interspecies Comparison

**FIGURE 7:**
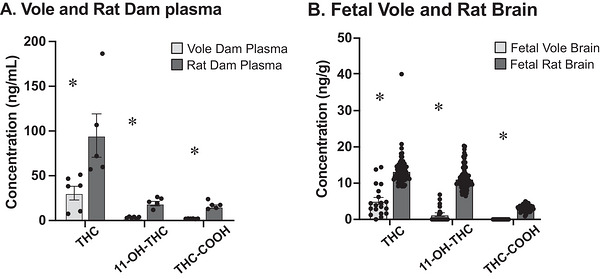
Direct comparison of THC concentration in adult (A) and fetal (B) rats and voles. (a) Plasma THC, 11‐OH‐THC, and THC‐COOH concentrations were all significantly higher in rat dams (dark gray) than in vole dams (light gray). (b) Brain THC, 11‐OH‐THC, and THC‐COOH concentrations were all significantly higher in rat fetal brains (dark gray) than in vole fetal brains (light gray). The asterisk (*) indicates that the value is significantly different between species.

A Wilcoxon rank‐sum test revealed significantly higher plasma THC concentrations in rat dams compared to prairie vole dams (*W* = 36; *p* = 0.005075; Figure 7A). For the metabolites of THC in plasma, the 11‐OH‐THC concentration between dosed rat dams and dosed prairie vole dams was significant (*W* = 36; *p* = 0.005075). The THC‐COOH concentration for the dams of both species was also found to be significantly different (*W* = 36; *p* = 0.005075).

Fetal brain THC concentrations were found to be significantly higher in THC‐exposed fetal rat brain tissue compared to THC‐exposed fetal voles (*W* = 1569; *p* = 6.045 × 10^−5^; Figure 7B). After running a Wilcoxon rank‐sum test for the dosed fetal brains of the rats and prairie voles, the metabolites also showed a significant species difference in the concentration of 11‐OH‐THC (*W* = 2039; *p* = 9.124 × 10^−14^) and THC‐COOH (*W* = 2040, *p* = 6.601 × 10^−14^).

### 3.4 Additional Prairie Vole Results

#### 3.4.1 Prairie Vole Pregnant Dam Brain Versus Fetal Brain

**FIGURE 8:**
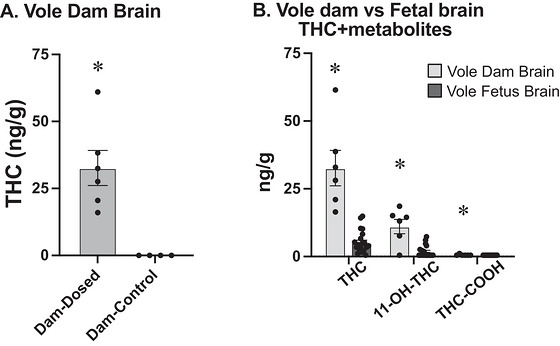
(a) Concentration of THC in the brains of dosed dams (left) and vehicles (right). The dosed dam brains were significantly higher than the vehicle dam brains. (b) Concentration of THC (left), 11‐OH‐THC (middle), and THC‐COOH (right) in the brains of prairie vole dams and fetal prairie voles. The concentration for the dams was significantly higher than that for the fetuses for THC and both its metabolites. The asterisk (*) indicates that the value is significantly different from all other groups.

The Shapiro–Wilk test revealed a normal distribution of THC concentrations in dam prairie vole brains between vehicle and dosed dams (*W* = 0.87898, *p* = 0.127). A Wilcoxon rank‐sum test showed a significant difference between dosed dam THC concentrations and vehicle dam THC concentrations for the brain (*W* = 24; *p* = 0.01142; Figure 8A). Maternal brain THC concentrations were found to be significantly higher than fetal THC levels in a linear mixed‐effects model accounting for individual dam variability (estimate = 24.6, *p* = 1.05 × 10^−9^; Figure 8B). There was also a significant difference in both the metabolites of THC in relation to dosed dam brain and dosed fetal brain, 11‐OH‐THC (estimate 8.775, *p* = 6.53 × 10^−8^) and THC‐COOH (estimate 0.106198, *p* = 0.0439).

#### 3.4.2 Prairie Vole Adult Brain and Plasma

**FIGURE 9:**
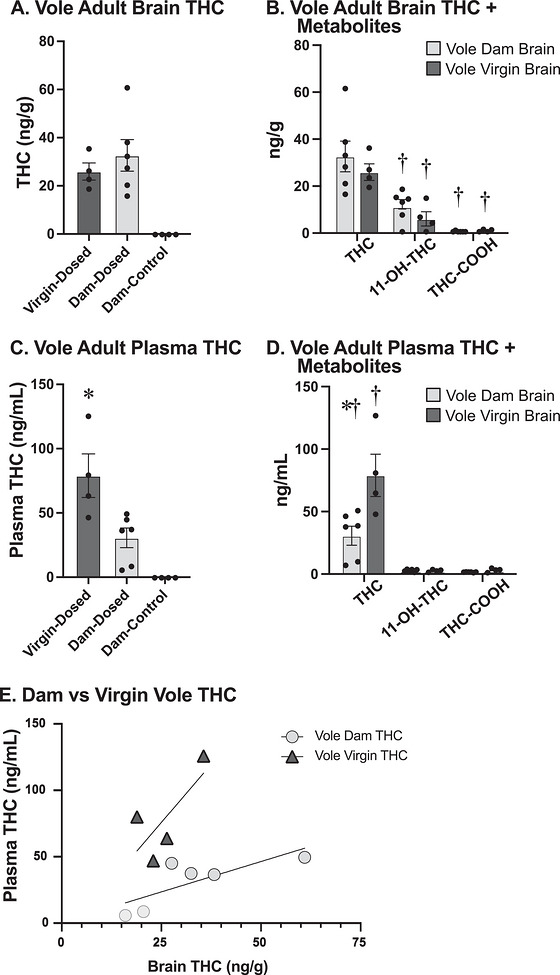
(a) Concentration of THC in the brains of dosed virgin female voles (left), dosed dams (center), and vehicle dams (right). The brains of the dosed dams contained significantly higher concentrations of THC than those of control dams. (b) Concentration of THC (left), 11‐OH‐THC (middle), and THC‐COOH (right) in the adult brains of prairie vole dams and female virgins. There was a significant difference in THC and metabolite concentrations within a group, but no significant difference between the dam and virgin female dosed brains. (c) The concentration of THC in the plasma of dosed virgin female voles (left), dosed dams (center), and control dams (right). The plasma of dosed dams contained significantly lower concentrations of THC than virgin females and significantly higher concentrations of THC than control dams. (d) THC and metabolite concentrations in the plasma of dosed vole dams. 11‐OH‐THC concentration (center) and THC‐COOH concentration (right) were both significantly lower than THC concentration (left) in the plasma of dams. There was no significant difference between 11‐OH‐THC and THC‐COOH. (e) Correlation between brain (*x*‐axis; ng/g) and plasma (*y*‐axis; ng/mL) THC concentrations in dams (clear circles) and virgin females (black triangles). The asterisk (*) indicates a significant difference between dams and virgin females. The plus symbol (+) indicates a difference in THC and metabolite concentrations within a group.

A subsequent Wilcoxon rank‐sum test did not reveal a sex difference between male and female virgin prairie voles for brain THC concentration (*W* = 13; *p* = 0.1939) or for plasma THC concentration (*W* = 7; *p* = 0.8852). Since there were no sex differences, only female virgins were used for subsequent analyses because they more closely approximate the experimental subjects. No significant difference in brain THC concentrations was found between dosed dams and female virgins (*W* = 15; *p* = 0.594; Figure 9A). For the adult dam brains and virgin female brains, we also measured the two major metabolites of THC, 11‐OH‐THC and THC‐COOH, and found some significant differences between THC and its metabolites (Figure 9B). There was a significant difference between the dosed and vehicle dams for 11‐OH‐THC (*W* = 22, *p* = 0.03073), but not a significant difference between dosed and vehicle dams for THC‐COOH (*W* = 14, *p* = 0.5403). For the dosed dams, the concentrations of the metabolites were significantly lower when compared to THC brain concentrations for both 11‐OH‐THC (estimated −21.56 ng/g, *p* = 0.00197) and THC‐COOH (estimated −32.535 ng/g, *p* = 4.67 × 10^−5^). For the dosed female virgins, the metabolite concentrations were also significantly lower than THC for both 11‐OH‐THC (estimated −19.925 ng/g, *p* = 0.000553) and THC‐COOH (estimated −25.511 ng/g, *p* = 9.10 × 10^−5^) compared to THC brain concentrations. Concentrations of THC and its metabolites did not differ significantly between dams and virgin females. However, plasma THC concentrations differed significantly between female virgin and dam prairie voles (*W* = 1; *p* = 0.02518; Figure 9C).

#### 3.4.3 Prairie Vole Virgin Plasma Metabolites

Additionally, for the virgin female plasma, we measured 11‐OH‐THC and THC‐COOH. For the dosed female virgins, the metabolite concentrations for plasma were also significantly lower than THC for both 11‐OH‐THC (estimated −77.415 ng/g, *p* = 0.000339) and THC‐COOH (estimated −76.940 ng/g, *p* = 0.000354; Figure 9D). The metabolite concentrations, 11‐OH‐THC and THC‐COOH, were not significantly different from each other for prairie vole virgin female plasma (*V* = 4, *p* = 0.8551). Concentrations of the metabolites of THC did not differ significantly between dams and virgin females, 11‐OH‐THC (*W* = 13, *p* = 0.9151) and THC‐COOH (*W* = 5.5, *p* = 0.1995).

#### 3.4.4 Prairie Voles Adult Brain Versus Plasma

A positive correlation, although not significant, was observed between plasma and brain tissue THC concentrations for the dams (*r* = 0.785, *p* = 0.065; Figure 9E) and female virgins (*r* = 0.737, *p* = 0.2635).

#### 3.4.5 Prairie Vole Fetal Sex

For the fetal dosed prairie voles, extracted fetal body tissues were used to determine the sex of the fetus. There was no significant difference in the THC concentration of fetal brains between male and female fetuses after accounting for litter size and litter effects (estimate 1.0714, *p* = 0.493; Figure 5E). There were also no significant differences between male and female fetal brains for 11‐OH‐THC after accounting for litter size and litter effects (estimate = 0.2697, *p* = 0.629). THC‐COOH was undetectable in all fetal prairie vole samples, so no statistical comparison could be run. Litter size was not a significant predictor in these analyses.

## 4 Discussion

These studies utilized established e‐cigarette vapor technology protocols (Taffe et al. 2021; Nguyen et al. 2016, 2018; Javadi‐Paydar et al. 2019) to deliver THC to pregnant vole and rat dams. While previous studies have utilized this translationally relevant delivery in rats (Ginder et al. 2024; Freels et al. 2020; Preteroti et al. 2023; Baglot et al. 2022), we are unaware of comparable studies in voles.

In this series of experiments, we demonstrated the efficacy of prenatal dosing of rodents with inhaled THC. We established that THC exposure within a vapor chamber will enter the maternal blood in both species and that THC will also cross the placental barrier into fetuses. Furthermore, the presence of THC metabolites in fetal brain tissue demonstrates that the THC is actively metabolized by the fetus. Finally, we demonstrated that there are species‐specific differences in the concentration of THC in both the dams and offspring of rats and prairie voles. Together, these results establish the vapor chamber as a reliable method of prenatal THC exposure and demonstrate the importance of carefully choosing the appropriate model species for a research question.

Prior studies have shown that the primary psychoactive compound in marijuana, THC, crosses the placental barrier quickly, whereas its primary metabolite (THC‐COOH) does not (Bailey et al. 1987). Our data support this idea, as the concentration of THC‐COOH was significantly lower than that of THC for both the fetal prairie voles and fetal rats. The placenta appears to reduce fetal exposure to marijuana, as evidenced by research in various animal models, which has documented that fetal THC concentrations are consistently lower than maternal concentrations (Behnke and Smith 2013). Our findings in prairie voles corroborate these earlier studies, demonstrating significantly higher THC concentrations in maternal brain tissue compared to fetal brain tissue. This difference suggests that while THC does cross the placental barrier, there may be additional protective mechanisms in place that limit fetal exposure (Kumar et al. 2023). However, it is crucial to note that despite this reduction, THC levels in dosed prairie vole fetuses were still significantly higher than in vehicle controls, indicating that prenatal exposure does occur and could potentially impact fetal development. Another important finding was that, while there were no significant differences in brain THC concentrations between prairie vole dosed dams and female virgins, there was a significant difference between the plasma THC concentrations of female virgin and dam prairie voles. A possible explanation could be that the fetuses are absorbing some of the THC circulating in the blood of the dam prairie voles. A study by the Lo group (Shorey‐Kendrick et al. 2023) reported placental dysfunction and altered DNA methylation following prenatal THC exposure in rhesus monkeys, indicating the potential of additional effects of THC on the placenta directly. In rats, our results similarly showed significantly higher THC concentrations in dosed fetal brain tissue compared to vehicle controls. The absence of differences between left and right uterine horn locations and uterine position in both rats and voles supports the finding that fetal position does not significantly impact THC exposure. This finding is important for understanding the uniformity of THC distribution across multiple fetuses and may have implications for studying the effects of prenatal THC exposure on litter‐wide outcomes.

For this study, we chose a short exposure window for several reasons. First, we hoped to reduce the likelihood of embryonic resorption or fetal death due to maternal stress. Second, we chose to target a period when the endocannabinoid system was present and active within the developing brain, and the brain regions involved in social behavior have gone through neurogenesis. Third, we wished to see whether a short exposure would result in effects on the fetus or whether chronic exposure was needed. Finally, this exposure time (embryonic day [E] 15–17 in voles and rats) roughly aligns with postconception days 58–76 in humans, which corresponds to the latter half of the first trimester (Workman et al. 2013). This time is significant because many pregnant people experience symptoms of morning sickness that can peak around this time, and relief of nausea is the second most commonly cited reason for using marijuana during pregnancy (Zaugg et al. 2024).

In rat embryos, neurulation does not occur until E9–10 (Morriss‐Kay 1981). Cannabinoid CB1 and CB2 receptor mRNA is first found in embryonic rat brains on E11 and increases in density through later gestation and the early postnatal period (Fride 2008). The hypothalamus undergoes neurogenesis from E10.5 to 18.5, but neuroendocrine cells become postmitotic at E12.5–14.5, when they migrate to their final destinations and begin to produce specific hormones (Maggi et al. 2015). By exposing the pregnant dams and embryos to THC at E15–17, we were able to target a time when cannabinoid receptors were present and hypothalamic neurons were beginning to form networks and connections. This developmental time point provided the greatest likelihood of disrupting the developing system at the earliest functional time point.

Our exposure paradigm is based on Baglot et al. (2022), which reported that repeated THC vapor inhalation in pregnant Sprague–Dawley rats resulted in higher THC concentrations in the maternal blood compared to the fetal brain, with fetal brain THC concentrations at about one‐third of dam blood levels. The authors concluded that the inhalation route produced lower fetal brain exposure than the more commonly used injection protocols. Similar to the Baglot et al. results, prairie vole fetal brain THC concentrations reached only a fraction of the prairie vole dam circulating levels, averaging ∼25% of maternal plasma concentrations. For the rats, the mean fetal brain THC concentrations corresponded to ∼ 15% of maternal plasma concentrations, also reflecting restricted dam–fetal THC transfer, but at a higher level. The difference in the Baglot et al. report of ∼30% rat fetal brain THC relative to maternal rat blood and our ∼15% rat THC ratio could be due to a variety of slight variations in procedures. It is important to note that Baglot et al. exposed dams to THC for 12 consecutive days and collected samples 15 min after the final dose, whereas our dams received 3 days of exposure and samples were collected immediately after the final session, which may contribute to the lower ∼15% ratio observed in our rat cohort. Certain methodological details, including whether whole blood or plasma was analyzed or whether the fetal brains were assayed as a whole‐brain tissue or a specific region, could also have contributed to the differences. Potential variation in the way the THC was quantified may further explain the fetal–dam ratios observed across studies. Our observed average rat dam plasma concentration (∼88 ng/mL) was also higher than that of Baglot et al. (∼65 ng/mL) and other reported values in studies focusing on motor effects (∼20 ng/mL; Hussain et al. 2022; Breit et al. 2020, 2022), which may be attributable to differences in sampling time, as those studies also collected samples 15–20 min postexposure.

The differences in THC levels between species deserve a deeper investigation, and there are several possible factors that may contribute to those differences. All subjects were administered the same dose of THC solution with the same number of puffs over the same amount of time. However, the size of the exposure chamber and air flow rate differed between species. Each exposure chamber was just large enough to fit a single cage so as to minimize the volume of space impacted by the THC vapor. The air flow rates were calibrated to maintain the same atmospheric pressure in each chamber. These variations could have contributed to the species differences reported here.

Another factor that may contribute to species differences in THC levels is the physical differences between rats and voles. The exact body composition of rats and voles varies dramatically based on diet, stress level, sex, and age. Generally, healthy adult voles and rats that are fed on standard lab chow will have a body composition that is ∼20% fat (Rose et al. 1998; Seelke et al. 2018). Adult rats are five to 10 times the size of adult voles, and every aspect of their bodies is allometrically scaled, so that even though they have similar percentage body fat, rats will have larger masses of fat. Since THC is lipophilic, it is possible that a greater amount of THC was bound to the adipose deposits in rats. Similarly, the volume of air in a single inhalation, also known as *tidal volume*, scales allometrically (Bide et al. 2000). Since rats have a larger tidal volume, each inhalation has the potential to bring in a larger amount of THC from the air.

Our use of an aerosolized administration chamber for THC administration represents a unique methodological process. This approach more closely mimics human consumption patterns, particularly given the increasing popularity of vaping among cannabis users. By standardizing this method across two rodent species, we provide a more translational model for studying the effects of prenatal cannabis exposure. While our study provides valuable insights into the distribution of THC in maternal and fetal tissues, it also highlights the need for further research. Future studies should investigate the long‐term developmental consequences of the observed THC exposure, particularly focusing on neurobehavioral outcomes (Halbout et al. 2023). Additionally, exploring the mechanisms behind the species differences in THC concentrations could provide valuable insights into the factors influencing prenatal THC exposure. The interspecies comparison revealing higher THC concentrations for rats compared to prairie voles for both fetal brain tissue and dam plasma is particularly intriguing. This difference could be attributed to various factors, including species‐specific placental structure, metabolism, or distribution of THC. Furthermore, even animals that are closely related, such as those in the family Muridae of the class Rodentia, show different patterns of development, anatomy, and behavior (Krubitzer et al. 2011). In this experiment, we chose to examine rats and prairie voles due to their specific behaviors that are seen in humans, but not commonly expressed in other laboratory rodents: juvenile play and pair bond formation. Our ultimate goal is to understand how prenatal exposure to THC impacts the development of complex social behaviors later in life, and the well‐established social behaviors exhibited by rats and prairie voles provide an excellent experimental model that will help us extrapolate findings to human scenarios. Both species had plasma THC concentrations that are within the range of human THC plasma concentrations, with plasma THC concentrations reaching ∼18–110 ng/mL in humans (Schwope et al. 2011). We have, however, been unable to find any specific citations that addressed plasma THC concentrations in pregnant humans. Thus, we can only hypothesize that the THC concentrations seen in this study would serve as a precise match for values in pregnant humans.

In conclusion, our study shows that prenatal THC exposure via aerosolized administration leads to significant concentrations of THC in the fetal brain of both prairie voles and rats, although these levels are lower compared to those found in dam tissue. The standardized aerosolized administration protocol used in this study provides a robust method for future investigations into the effects of prenatal cannabis exposure. If the use of cannabis continues to increase as it has in past years, including among pregnant women, understanding the implications of prenatal exposure becomes increasingly critical (Young‐Wolff et al. 2024; Mattingly et al. 2024).

## Conflicts of Interest

The authors declare no conflicts of interest.

## Data Availability

The data that support the findings of this study are available from the corresponding author upon reasonable request.
